# Proposal for Managing Cancer‐Related Insomnia: A Systematic Literature Review of Associated Factors and a Narrative Review of Treatment

**DOI:** 10.1002/cam4.70365

**Published:** 2024-11-25

**Authors:** Yoshinori Saeki, Yukiyoshi Sumi, Yukinori Ozaki, Mari Hosonaga, Yoshiyuki Kenmotsu, Takuma Onoe, Ken Yamaguchi, Yoshihisa Matsumoto

**Affiliations:** ^1^ Department of Palliative Therapy The Cancer Institute Hospital of Japanese Foundation for Cancer Research Tokyo Japan; ^2^ Department of Psychiatry Shiga University of Medical Science Otsu Shiga Japan; ^3^ Breast Medical Oncology The Cancer Institute Hospital of Japanese Foundation for Cancer Research Tokyo Japan; ^4^ Department of Respiratory Medicine Kin‐Ikyo Chuo Hospital Sapporo Japan; ^5^ Department of Medical Oncology Hyogo Cancer Center Akashi Japan; ^6^ Department of Gynecology and Obstetrics, Graduate School of Medicine and Faculty of Medicine Kyoto University Kyoto Japan

**Keywords:** cancer, insomnia, psycho‐oncology, review, sleep disturbance

## Abstract

**Objective:**

Insomnia is common in patients with cancer. It has a multifactorial etiology that may include the disease process, adverse effects of anticancer therapies, and/or an association with other comorbidities. The purpose of this review was to identify risk factors for insomnia and suggest optimal management strategies.

**Methods:**

We conducted a systematic literature review to elucidate the risk factors for insomnia and sleep disturbances in patients with solid tumors. The effects of sleep medications in this population were also described.

**Results:**

A total of 75 publications were evaluated, including those on breast, lung, gynecologic, brain, head and neck, gastrointestinal, prostate, thyroid, and mixed cancers. We classified the factors related to insomnia or sleep disturbance in cancer into four categories: (1) patient demographic characteristics (e.g., age, marital or socioeconomic status); (2) mental state (e.g., depression or anxiety); (3) physical state (e.g., fatigue, pain, or restless legs syndrome); and (4) anticancer treatment‐related (e.g., use of chemotherapy, opioids, or hormone therapy). Overall, literature on the pharmacologic treatment of insomnia is extremely limited, although some efficacy data for zolpidem and melatonin have been reported.

**Conclusions:**

Demographic characteristics, physical and mental distress, and anticancer treatments are all risks for insomnia in patients with cancer. The limited evidence base for pharmacologic therapy in this patient population means that healthcare professionals need to implement a comprehensive and multidisciplinary pathway from screening to management.

## 1 Introduction

It has been estimated that, globally, at least one in five people develops cancer each year, and the number of cases is set to increase by more than 50% over the next 15 years as the worldwide population continues to grow and age [1]. Reports have shown that sleep disturbances such as insomnia are frequently reported by individuals with cancer, with incidences ranging from 30% to 95% depending on the type of cancer, and on the study design and measuring instruments used [2, 3]. Patients with cancer can develop concomitant sleep disturbances at any time, from the onset of the disease, during treatment, and as their cancer progresses [3]. Cancer‐related insomnia (characterized as delayed onset, poor maintenance, and reduced duration of sleep) is a contributing factor to decreased quality of life in affected patients due to daytime fatigue and sleepiness and impaired wellbeing and performance [3, 4, 5].

The causes of cancer‐related insomnia are multifactorial and include sleep disturbances (reported to be associated with multiple types of cancer) [2] and cancer therapies (including surgery, chemotherapy, and radiotherapy) [6]. Other comorbid symptoms often observed in patients with cancer (such as depression, anxiety, fatigue, pain, obstructive sleep apnea (OSA), and restless legs syndrome [RLS]) can also cause and exacerbate sleep disturbances, making it difficult to develop a standardized management strategy [3]. Symptoms of insomnia may also manifest in other more general sleep disorders such as OSA and RLS; in recent times, an increasing number of publications have reported on the association between cancer and sleep‐related breathing, particularly OSA, especially among patients with head and neck cancer or lung cancer [7, 8]. In terms of treating insomnia in patients with cancer, although the efficacy of non‐pharmacologic therapies has been evaluated in multiple studies and meta‐analyses [9, 10, 11], evidence on the efficacy of pharmacologic options is limited [12]. There is also a lack of clear guidance on which patients may be at higher risk for insomnia due to their background characteristics, and the decision of which patients should be referred to an insomnia specialist is left to the experience and judgment of individual physicians.

To better manage insomnia in patients with cancer, it is important to obtain a complete picture of the factors involved to help identify those patients at greater risk of developing insomnia. In this study, we conducted a systematic literature review of publications reporting the prevalence of insomnia or sleep disturbances in patients with cancer and the association between insomnia and patient background characteristics. The effects of sleep medications in this population were also assessed and described narratively. The aim was to identify risk factors for insomnia or sleep disturbance and to suggest optimal management strategies for affected patients.

## 2 Methods

### 2.1 Search Strategy and Data Extraction

We conducted a literature search for published articles reporting the prevalence of insomnia or sleep disturbances and their risk factors in patients with cancer, following Preferred Reporting Items for Systematic Reviews and Meta‐Analyses 2020 guidelines [13]. The databases used were PubMed and Scopus, and the search query implemented was based on “Insomnia OR sleep disturbance OR sleep disorder OR hypnotics” AND “Cancer OR tumor” (see Data S1 for full details). There were no restrictions by language or country; however, because chemotherapy regimens for cancer have changed considerably in recent years, the search was limited to studies in the most recent 10‐year period (2012–2022). Hematologic malignancies were not included in this systematic review because their treatment strategies differ notably from those of solid tumors with respect to surgery and hematopoietic stem cell transplantation.

Following the initial identification step, the resulting articles were screened (Figure S1). We included studies that evaluated the frequency of insomnia in solid tumors and also those that reported factors statistically significantly associated with insomnia or sleep disturbance. Exclusion criteria included studies that did not use diagnostic criteria for insomnia, studies that did not use questionnaires related to insomnia or sleep disturbance, and case series. Studies that only targeted cancer survivors were also excluded, as our intent was to focus specifically on patients actively undergoing treatment for cancer.

The full‐text versions of the remaining articles were screened for eligibility. During this process, only studies that used well‐validated and empirically supported questionnaires (such as the Pittsburgh Sleep Quality Index [PSQI], Insomnia Severity Index [ISI], or Athens Insomnia Scale [AIS]) to assess insomnia or sleep disturbance, or studies based on diagnostic criteria such as the International Classification of Sleep Disorders (ICSD)‐2 or −3 and the Diagnostic & Statistical Manual of Mental Disorders (DSM)‐IV or ‐V were included. In addition, among the studies that examined insomnia in cancer, we included only those articles that specifically evaluated risk factors for insomnia. For each selected article, we extracted the information into tables summarizing the cancer therapy used (surgery, radiotherapy, and chemotherapy), the assessment scale and prevalence of insomnia or sleep disturbance, and factors that were reported to be statistically significantly associated with insomnia or sleep disturbance.

### 2.2 Analysis of Pharmacologic Therapies for Insomnia

We conducted a narrative review, evaluating data from studies that reported the effects of sleep medications for insomnia in patients with solid tumors, because a previous analysis of pharmacologic therapy for insomnia in patients with cancer was limited and did not include recent medications such as dual orexin receptor antagonists (DORAs) [12]. Non‐pharmacologic therapies were not included in this analysis as they have been evaluated in previous reports [11, 14, 15]. We chose to produce a narrative review instead of a systematic review, primarily because the evidence for the use of pharmacologic therapy to manage insomnia in patients with cancer was anticipated to be extremely heterogeneous (i.e., wide variability in characteristics such as the study setting, cancer type and stage, type of anticancer treatment, and whether the patient was considered to have terminal disease). Furthermore, because of the expected variability in the type and stage of the underlying cancer and the different anticancer treatments administered, we did not conduct a data integration according to categories of sleep medications.

## 3 Results

### 3.1 Prevalence of Insomnia or Sleep Disturbance in Patients With Cancer

Our literature search identified a total of 75 studies for analysis, including 23 breast cancer, seven lung cancer, four gynecologic cancer, four brain cancer, four head and neck cancer, nine gastrointestinal cancer, six prostate cancer, one thyroid cancer, and 17 mixed cancer types. Data extracted from these studies are reported in Table 1. The prevalence of insomnia or sleep disturbance among patients with cancer ranged from 14.8% to 81.5%.

**TABLE 1:** Prevalence of insomnia and factors statistically significantly related to insomnia in patients with cancer.

Cancer type	Study	Events	Sample size	Insomnia prevalence	Cancer status	Cancer stage	Surgery	RT	CT	Insomnia or sleep disturbance definition	Factors significantly related to insomnia
Breast	Alsharif [16]	29	101	28.8%	Undergoing treatment	Mixed	Yes	—	Undergoing CT	PSQI (> 5)	Fatigue, age, marital status, income, employment, country
	Bauml [17]	67	203	33.2%	Early BC	Stages I—III (0, I/II/III: 47%/40.5%/12.5%)	—	—	AI 98%, CT 61.7%	ISI (> 14)	Joint pain, fatigue
	Bean [18]	366	460	79.6%	—	Stage I, II/III, IV: 83.3%/16.7%	90.8%	37%	CT 53%, HT 64%	WHIIRS (≥ 9)	Marital status, employment, younger age, depression, anxiety, chronic life stress
	Berger [19]	690	1302	53%	Treatment—naïve	Stage I—II 78.6%	—	40.1%	CT 48.6%, HT 36.3%	PSQI (> 5)	Younger age, physical activity, fatigue
	Colagiuri [20]	1738	3002	57.9%	—	Lymph node 18.1%	100%	43.3%	44.5%	PSQI (> 5)	Depression, poor physical functioning, older age, anxiety, smoking, lumpectomy
	Desai [21]	207	413	50.2%	Adjuvant CT	Stage 0—III (I/II/III: 40.4%/47.2%/12.4%)	100%	69.7%	AI 100%; CT 61.7%	ISI (> 7)	Joint pain, hot flash, anxiety, depression
	Fakih [22]	30	52	Prior CT 36%, current CT 58%	Early, CT—naïve	Stage I—III	—	—	Prior or current CT	PSQI (≥ 5)	CT
	Fekih—Romdhane [23]	33	50	66%	Post—surgery	Stage 0/I/II/III: 0%/10%/58%/28%	100%	64%	CT 88%, HT 64%	PSQI (> 5)	Depression, hopelessness
	Fleming [24]	80	173	46%	Non—metastatic/ CT—naïve	Stage I/II/III/IV: 38.2%/26.0%/19.1%/6.4%	100%	93.6%	CT 46.2%, HT 84.4%	ISI (> 7)	CT
	Fontes [25, 26]	302	502	60.2%	Treatment—naïve	Stage 0/I/II/III/IV: 6.8%/46.8%/30.9%/14.9%/0.6%	0% (100% at 1y)	73.6%	59.3%	PSQI (> 5)	Anxiety, depression, RT, neuropathic pain
	Hajj [27]	31	112	27.6%	Current/post—treatment	Metastatic 7.1%	—	—	100%	ISI (≥ 15)	Depression
	Ho [28]	91	197	46%	—	Stage I—III	56.4%	70.1%	78.1%	PSQI (≥ 8)	Anxiety, depression, fatigue, pain
	Jung [29]	40	198	20.2%	Treatment—naïve	Stage I/II/III: 29.3%/58.6%/12.1%	100%	—	—	ISI (≥ 10)	CINV
	Liu [30]	59	97	61%	Treatment—naïve	Stage I/II/III: 30.2%/50.0%/19.8%	86.5%	—	Undergoing CT	PSQI (> 5)	Fatigue, total nap time
	Mansano—Schlosser [31]	92	156	58.9%	Before surgery	Stage I, II/III, IV: 78.7%/21.3%	Planned	2.6%	25.5%	PSQI (> 5)	Pain, menopause, depression

	Mete Civelek [32]	65	111	58.6%	Lymphedema	Stage I/II/III: 11.7%/42.7%/45.9%	100%	92.8%	CT 96.4%, HT 90.1%	PSQI (> 5)	Depression, older age
	Pedersini [33]	80	160	50.0%	Early BC	Stage I—III	100%		33%	ISI (> 7)	RLS
	Peoples [34]	85	573	14.8%	Post—RT	—	99%	100%	CT 87.5%, HT 31.9%	MDASI (≥ 4)	Younger age, marital status, mastectomy, CT, depression, anxiety, pain, fatigue
	Ren [35]	NA	749	NA	—	Stage I/II/III: 11.3%/62.2%/26.4%	100%	—	0%	NS	Neuroticism, anxiety, optimism
	Sanford [36]	53	80	65.8%	CT—naïve	Stage I/II/III: 10%/73%/16%	100%	57%	CT 100%, HT 44%	PSQI (≥ 5)	Fatigue, depression, vasomotor/endocrine symptoms
	Wang [37]	55	108	51%	Pre— or post—surgery	Localized	100%	—	—	PSQI (≥ 5)	Postoperative pain scores
	Weng [38]	303	448	67.6%	—	Metastatic 3.2%	18.6%	56%	69.4%	PSQI (> 5)	Psychologic distress, pain, depression, stress
Zhu [39]	129	329	Before CT 27.0%, current CT 32.5%, post—CT 39.2%	Before/current/ post—CT	Metastatic 9.7%	22.8%	—	100%	PSQI (> 8)	Anxiety, older age
Lung	Bülbül [40]	603	1245	48.40%	—	Stage I, II/III, IV: 15.9%/78.6%; metastatic 52.9%	—	—	—	DIMS yes	Pain, dyspnea, anxiety
	Halle [41]	181	264	Baseline 60.9%, 1 month post—surgery 68.5%	Pre— or post—surgery	Stage I/II/IIIa/IIIb/IV: 62.6%/18.4%/16.4%/0.4%	100%	—	—	GSDS (≥ 43)	Younger age, pain/psychotropic medication use, comorbidity score
	He [42]	55	98	56.1%	Advanced (planned CT)	Advanced (Stage III/IV: 18.4%/81.6%)	—	—	—	PSQI (> 5)	Anxiety, depression
	Lou [43]	80	128	62.5%	Advanced	Metastatic 75.0%	—	23.4%	93.8%	PSQI (≥ 5)	QOL
	Mercadante [44]	106	182	48.5%	Advanced	Advanced	—	5.0%	25.1%	AIS (≥ 6)	KPS score, pain, nausea, anxiety, depression
	Nishiura [45]	28	50	56%	—	Mixed	—	—	62%	AIS (≥ 6)	HADS score, fatigue, pain, QOL
	Papadopoulos [46]	69	119	58.2%	Planned chemotherapy	Stage I, II/III/IV: 2.5%/20.2%/77.3%; brain metastasis 15.1%	3.4%	16.1%	Undergoing CT	PSQI (> 5)	Anxiety, stress, positive coping, fatigue, low PS
Gynecologic	Tian [47]	49	76	Before/After adjuvant CT, 52.63/64.47%	Cervical	Stage I/II: 44.7%/55.2%	100%	48.68%	100%	PSQI (> 5)	Depression, anxiety
	Li [48]	52	95	55%	Endometrial	Stage I/II/III/IV: 83.2%/2.1%/10.5%/2.1%	97.9%	9.5%	4.2%	PSQI (> 5)	Fatigue
	Clevenger [49]	122	173	70.7%	Ovarian cancer with pelvic mass	Stage I/II/III/IV: 20.7%/6.5%/62.5%/10.3%	100%	—	—	PSQI (> 5)	Depression, pain medication, QOL
	Pozzar [50]	189	232	81.5%	Ovary 57.0%, uterine 29.8%, other 13.2%	Metastatic 72.8%	—	—	100%	GSDS (≥ 43)	Depression, anxiety, fatigue, pain
Brain	Willis [51]	73	119	61.5%	—	High grades (3 or 4) 61.3%	—	—	—	PSQI (> 5)	Fatigue
	King [52]	81	424	19%	—	High grades (3 or 4) 74%	—	24%	—	MDASI—BT, sleep disturbance item (≥ 5)	Younger age, KPS score, tumor progression on MRI, active corticosteroid use
	Jeon [53]	43	81	53%	—	High grades (3 or 4) 73%	93%	—	43%	PSQI (> 5)	Fatigue, KPS score, anxiety, depression, pain
	Huang [54]	135	358	37.7%	—	—	100%	36%	—	PSQI (> 7)	Older age, postoperative duration, CT
Head and neck	Santoso [55]	246	560	44%	—	High grades (3 or 4) 58%	—	—	—	PSQI (≥ 5)	Younger age, female, higher passive coping style, oral pain, less sexual interest and enjoyment
	Santoso [56]	135	412	32.7%	—	High grades (3 or 4) 56.3%	23%	32%	45%	PSQI (> 5)	Female, painkillers prior to treatment, anxiety
	Mo [57]	33	51	64.7%	Newly diagnosed	Stage III/IV: 84.3%	—	100%	100%	PSQI (> 5)	RT, pre—RT poor sleep quality
	Zubair [58]	112	170	65.9%	—	—	100%	15%	14%	PSQI (> 5)	Psychiatric morbidity, surgery
Gastrointestinal	Bonhof [59]	155	340	46%	—	High grades (3 or 4) 51%	97%	15%	SPN:63%, MPN:43%	PSQI (> 5)	Sensory/motor peripheral neuropathy
	Lin [60]	260	405	64.2%	—	—	—	—	100%	GSDS (≥ 43)	Anxiety, depressive symptoms, fatigue, pain, lower levels of attentional function, QOL
	Lv [61]	not reported	352	not reported	—	—	57.1%	—	42.9%	ISI (> 8)	Psychologic distress
	Zhu [62]	78	115	67.8%	—	clinical stage II + III 95%	100%	—	90%	PSQI (≥ 11)	Number of CT cycles, fatigue, depression
	Sun [63]	166	434	38.2%	—	High grades (3 or 4) 36.9%	0%	—	—	AIS (≥ 6)	Pain, anxiety
	Yoshikawa [64]	70	139	50%	—	High grades (3 or 4) 86%	—	—	100%	PSQI (> 6)	Older age, second—line CT
	Coles [65]	153	613	25%	—	High grades (3) 39.8%	91.4%	—	54.6%	PROMIS sleep disturbance T—score (≥ 57)	Pain, anxiety, fatigue, and number of comorbidities
	Steel [66]	173	294	59%	—	—	—	—	—	PSQI score (7.6)	Fatigue, pain, anxiety, depression, QOL
	Innominato [67]	202	361	56%	—	—	—	—	100%	EORTC QLQ—C30, sleep trouble scale (≥ 33.3)	Progression and poor treatment response
Prostate	Galvin [68]	84	142	59.2%	—	—	—	—	—	ISI (≥ 8)	Anxiety, depression, fatigue, sleepiness
	Mondal [69]	37	74	50%	—	Gleason score: 8—9 35%	—	—	—	PSQI (> 5)	ADT
	Garland [70]	44	112	39%	—	Gleason score: 8—10 47%	96%	—	—	ISI (> 8)	Fatigue, depressive symptoms
	Sharpley [71]	59	96	61%	undergoing treatment 35.7%	—	8%	29.5%	10.2%	ISI (> 8)	Cognitive depression, having a clear mind
	Gonzalez [72]	39	78	50%	—	Gleason score: 8—10 26%	100%	28%	—	ISI (≥ 8)	ADT, nocturia, hot flashes
	Koskderelioglu [73]	52	106	49%	—	Gleason score: > 8 64.5% High grades (3 or 4) 64.6%	—	—	—	PSQI (≥ 5)	Fatigue, depression
Thyroid	He [74]	88	162	54.32%	—	—	100%	—	100%	PSQI (> 5)	Metastasis
Mixed	Abebe [75]	142	264	53.79%	Breast 44.3%, GI 16.3%, gynecologic 9.8%, HNC 8.7%, other	Stage III/IV: 59.8%	44.7%	8.8%	100%	PSQI (> 5)	Income, fatigue, pain, poor social support, anxiety, depression
	Asok [76]	108	164	65.9%	Breast 37.8%, GI 18.9%, lung 11.6%, gynecologic 10.4%, other	Stage III/IV: 74.4%	14.6%	17.1%	53%	PSQI (≥ 5)	Anxiety, depression, pain
	Cha [77]	95	208	45.7%	Breast 43.8%, GI 16.8%, liver 3.8%, gastric 16.3%, other	Stage III/IV: 39.9%	76.9%	45.6%	CT 58.2%, HT 20.7%	ISI (≥ 15)	Depression
	Edmed [78]	212	351	59%	Breast 94.7%, other 5.3%	Localized	Yes	100%	Undergoing CT	PSQI (≥ 5)	Older age, marital status, menopausal symptoms, pain, education
	Garousi [79]	167	250	66.8%	Breast 45.2%, GI 24.0%, other	Metastatic 34.8%	—	—	Undergoing CT	PSQI (> 5)	Physical dysfunction
	George [80]	164	256	64%	GI 25%, lung 11%, gynecologic 9%, sarcoma 6%, melanoma 6%, other	Early stage	—	54%	100%	PSQI (> 5)	Fatigue, symptom burden, mood disturbance
	Hoang [81]	91	213	42.8%	Breast 27.7%, lung 19.2%, GI 17.8%, other	Stage III/IV: 55.9%	66.2%	—	Undergoing CT	ISI (≥ 15)	Anxiety, depression
	Mansano—Schlosser [82]	88	140	62.9%	Digestive 52.8%, breast 52.1%	Advanced	73.6%	—	Undergoing CT	PSQI (≥ 5)	Pain
	Momayyezi [83]	103	149	69.3%	Intestinal/colorectal 27.6%, breast 20.1%, esophageal/gastric 11.4%, leukemia/lymphatic 16.1%, other	—	66.5%	32.2%	100%	PSQI (> 5)	Fatigue
	Saini [84]	101	173	58.8%	Colorectal 32.4%, breast 17.3%, prostate 7.5%, other	Metastatic 63.6%	—	—	Undergoing CT (cycle 3)	PSQI (≥ 5)	Sex, depression, anxiety, RLS, HADS

	Savard [85]	406	728	BC 66.2%, PC 36.9%	Breast 465, prostate 263	Localized	Planned	85.6% (BC) 4.2% (PC)	CT 54.1%, HT 72.0% (BC); CT 0.8%, HT10.4% (PC)	ISI (> 8)	BC: RT, CT
											PC: ADT
	Schieber [86]	200	405	49.4%	Breast 30.6%, prostate 14.7%, colorectal 13.7%, lung 8.4%, other	Localized 82.9%	—	—	Undergoing CT	ISI (> 7)	Sex, fatigue
	Strik [87]	48	107	44.9%	Breast 17.8%, brain 16.8%, colorectal 9.3%, pancreas 8.4%, other	—	—	—	90%	ISI (> 7)	Depression, anxiety, change of job
	Tejada [88]	998	1331	75.0%	Breast 40.3%, gastric 17.4%, GI 30.4%, lung 11.8%	Localized 32.5%	—	—	Undergoing CT	GSDS (total score ≥ 43, each item ≥ 3)	Younger age, KPS score, SCQ score, sex, employment, BMI
	Wang [89]	127	330	38.5%	Lung 29.1%, HNC 26.1, colorectal 19.1%, breast 16.0%, etc.	Localized 86.4%	49.4%	No	—	PSQI (> 7)	BMI, surgery
	Wang [90]	142	297	47.8%	Lung 23.2%, thyroid 24.2%, breast 20.2%, GI 32.3%	—	59.3%	—	—	PSQI (≥ 7)	Sex, anxiety
Harrold [91]	98	294	62%	breast (37.4%), colorectal (12.9%), lung (12.2%), Gynecologic (11.2), etc.	—	56.5%	41%	94.6%	ICSD—1, DSM—IV	Female, younger age (< 65 years), cancer subtype, alcohol consumption, HADS—depression/anxiety score ≥ 11

Abbreviations: ADT, androgen deprivation therapy; AI, aromatase inhibitor; BC, breast cancer; BMI, body mass index; CINV, chemotherapy‐induced nausea/vomiting; CT, chemotherapy; DIMS, difficulty initiating and maintaining sleep; DSM‐IV, Diagnostic and Statistical Manual of mental disorders, fourth edition; EORTC QLQ‐C30, European Organization for Research and Treatment of Cancer quality of life 30‐item core questionnaire; ESS, Epworth Sleepiness Scale; GI, gastrointestinal; GSDS, General Sleep Disturbance Scale; HADS, Hospital Anxiety and Depression Scale HNC, head and neck cancer; HT, hormone therapy; ICSD, International Classification of Sleep Disorders; ISI, Insomnia Severity Index; KPS, Karnofsky Performance Scale; MDASI, MD Anderson Symptom Inventory; MDASI‐BT, MDASI‐Brain Tumor; NS, not stated; PC, prostate cancer; PROMIS, Patient‐Reported Outcomes Measurement Information System; PS, performance score; PSQI, Pittsburgh Sleep Quality Index; QOL, quality of life; RLS, restless legs syndrome; RT, radiotherapy; SCQ, Social Communication Questionnaire; WHIIRS, Women's Health Initiative Insomnia Rating Scale.

Many (36/75 [48.0%]) of the studies included in our analysis used the PSQI, with a cutoff of > 5 or ≥ 5. When assessed according to a PSQI score > 5, the prevalence of insomnia or sleep disturbance was generally reported to be within the range of 29%–71%. Using more stringent PSQI cutoffs of ≥ 7, > 7, ≥ 8, or > 8 resulted in prevalence estimates ranging from 37.7% to 47.8%. The next most frequently used instrument was the ISI, using cutoffs of > 7, > 8, > 14, and > 15 (where scores of 8–14 reflect subthreshold insomnia). Insomnia or subthreshold insomnia prevalence assessed by ISI > 7 ranged from 45% to 50%.

The largest number of studies, including those with mixed cancer types, reported data from patients with breast cancer. We observed no notable differences between cancer types when comparing insomnia or sleep disturbance prevalence evaluated with PSQI > 5 (28.8%–67.6% for breast cancer, 56%–58% for lung cancer, 52.6%–70.7% for gynecologic cancer, 53%–61.5% for brain cancer, 32.7%–65.9% for head and neck cancer, 46% for gastrointestinal cancer, 50.0% for prostate cancer, 54.3% for thyroid cancer, and 53.8%–69.3% for mixed cancer types).

In the majority (44/75 [58.7%]) of the included studies, more than half of the patients were receiving chemotherapy. Of these, 38 studies used the PSQI to determine insomnia prevalence; using a PSQI cutoff of > 5, the prevalence of insomnia and impaired sleep quality in these 38 studies ranged from 52.6% to 67.6%, which was similar to the overall estimates.

### 3.2 Factors Statistically Significantly Associated With Cancer‐Related Insomnia

In our analysis, depression, anxiety, fatigue, and pain were frequently identified as factors statistically significantly associated with insomnia or sleep disturbance. Among the 75 studies evaluated, depression was reported to be a statistically significant factor in 31 (41.3%) studies (Table 1). Anxiety, fatigue, and pain were reported to be statistically significant factors in 26 (34.7%), 24 (32.0%), and 24 (32.0%) studies, respectively. In addition, other comorbidities, such as stress (psychologic distress), RLS, and dyspnea, were identified as being associated with insomnia. Chemotherapy, radiotherapy, surgery, and cognitive issues have also been found to be associated factors (Table 1).

### 3.3 Analysis of Pharmacologic Insomnia Treatments for Patients With Cancer

Among the 11 articles that evaluated the pharmacologic treatment of insomnia in patients with cancer (breast, brain, colorectal, or mixed), the most frequently used pharmacologic agents were melatonin and zolpidem (Table 2). Results indicated improvements in various aspects of sleep quality or efficiency, although it must be noted that most studies used questionnaires to rate sleep rather than objective measures such as sleep polysomnography (PSG) or actigraphy.

**TABLE 2:** Pharmacologic therapy for insomnia.

Cancer type	Study	Design	Patients with insomnia	No. patients	Stage, *n*	Surgery	Chemotherapy	Hypnotic	Insomnia/ sleep disturbance scale	Results
Breast	Chen [92]	RCT	Postmenopausal BC survivors	91 (melatonin, *n* = 46)	Stage 0—III	—	Completed active cancer treatment	Melatonin	PSQI	PSQI; significantly improved (*p* < 0.001 vs. placebo)
	Hansen [93]	RCT (secondary endpoints)	BC, scheduled surgery	54 (melatonin, *n* = 28)	—	Lumpectomy or mastectomy	CT, HT, RT	Melatonin	Sleep diary	Sleep efficiency (*p* = 0.02 vs. placebo), total sleep period (*p* = 0.03 vs. placebo)
	Innominato [94]	Prospective	Metastatic BC	32	—	—	HT, CT	Melatonin	L6, pAR, SFI	Improved objective sleep quality, sleep fragmentation and quantity, subjective sleep
	Madsen [95]	RCT	BC, perioperative	27	—	Perioperative	—	Melatonin	VAS for sleep, KSS	Improved sleep efficiency, wake after sleep onset, but no effects on VAS, KSS
	Palmer [96]	RCT	BC, adjuvant CT	36 (melatonin, *n* = 18)	—	Lumpectomy or mastectomy	Adjuvant	Melatonin	PSQI	PSQI; significantly improved (*p* < 0.001 vs. placebo)
Colorectal	Shahrokhi [97]	RCT	CRC undergoing CT	90 (zolpidem, *n* = 45, melatonin, *n* = 45)	—	—	CT	Zolpidem, melatonin	PSQI, GSOS	PSQI, GSOS: significantly improved with both hypnotics (*p* < 0.05)
Brain	Chang [98]	Prospective	Brain tumor resection	10	—	Resected	—	Zolpidem, trazodone	PSQI, ISI, SSS, ESS	PSQI, ISI, ESS: significantly improved
Mixed	Asok [99]	Retrospective	Solid tumor	34	—	—	—	Zolpidem	PSQI	PSQI components significantly improved (sleep latency, overall sleep quality, trouble staying awake during the daytime, trouble staying motivated to get things done)
	Jakobsen [100]	RCT	Advanced cancer (GI, *n* = 12; hematologic, *n* = 8; urologic, *n* = 7; others)	39 (zopiclone, *n* = 18)	Stage IV	—	—	Zopiclone	NRS, SOL, TST	Patient—reported sleep quality (NRS) and SOL: significantly improved (*p* = 0.021 and *p* = 0.045, respectively, vs. placebo)
	Kurdi [101]	RCT	HNC 54%, cervical 12%	50 (melatonin, *n* = 25)	Stage I/II, 25; Stage III/IV, 25	—	—	Melatonin	AIS	AIS scores: significantly improved (*p* < 0.05 vs. placebo)
	Terada [102]	Retrospective	Patients with cancer and delirium (HBP 35.7%, hematologic 21.4%, others)	14	—	—	—	Lemborexant	PRO	Improved 78.6%

Abbreviations: AIS, Athens Insomnia Scale; BC, breast cancer; CRC, colorectal cancer; CT, chemotherapy; ESS, Epworth Sleepiness Scale; GI, gastrointestinal; GSOS, Groningen Sleep Quality Scale; HBP, hepatobiliary pancreatic cancer; HNC, head and neck cancer; HT, hormone therapy; ISI, Insomnia Severity Index; KSS, Karolinska Sleepiness Scale; L6, average activity during 6 least active hours; NRS, numeric rating scale; pAR, probabilistic metric of activity fragmentation; PRO, patient‐reported outcome; PSQI, Pittsburgh Sleep Quality Index; RCT, randomized controlled trial; SFI, sleep fragmentation index; SOL, sleep onset latency; TST, total sleep time; VAS, visual analog scale.

Melatonin was evaluated for its efficacy in insomnia in several publications, but particularly in patients with breast cancer. Improvement in sleep quality based on objective and subjective measures has been observed, including two studies that showed statistically significant improvement in PSQI [92, 96]. Furthermore, a statistically significant improvement in AIS scores was also observed in a trial focusing on head and neck cancer [101]. The efficacy of zolpidem for insomnia has been evaluated in two studies: a comparative study with melatonin in patients with colorectal cancer [97], and a prospective study in patients with brain tumors [98]. There was one retrospective observational study of the DORA lemborexant, evaluating its efficacy for insomnia in patients with hepatobiliary pancreatic cancer and other cancer types [102]. In that study, lemborexant was reported to be effective in 11/14 (78.6%) patients with insomnia.

## 4 Discussion

The purpose of this study was to identify risk factors for cancer‐related insomnia and propose management strategies. A systematic literature review was conducted to identify articles reporting on insomnia or sleep disturbance in patients with cancer. A total of 75 articles were included, and although the definition of insomnia may have differed among the studies, the prevalence of insomnia or sleep disturbance among patients with cancer was consistently high.

**FIGURE 1:**
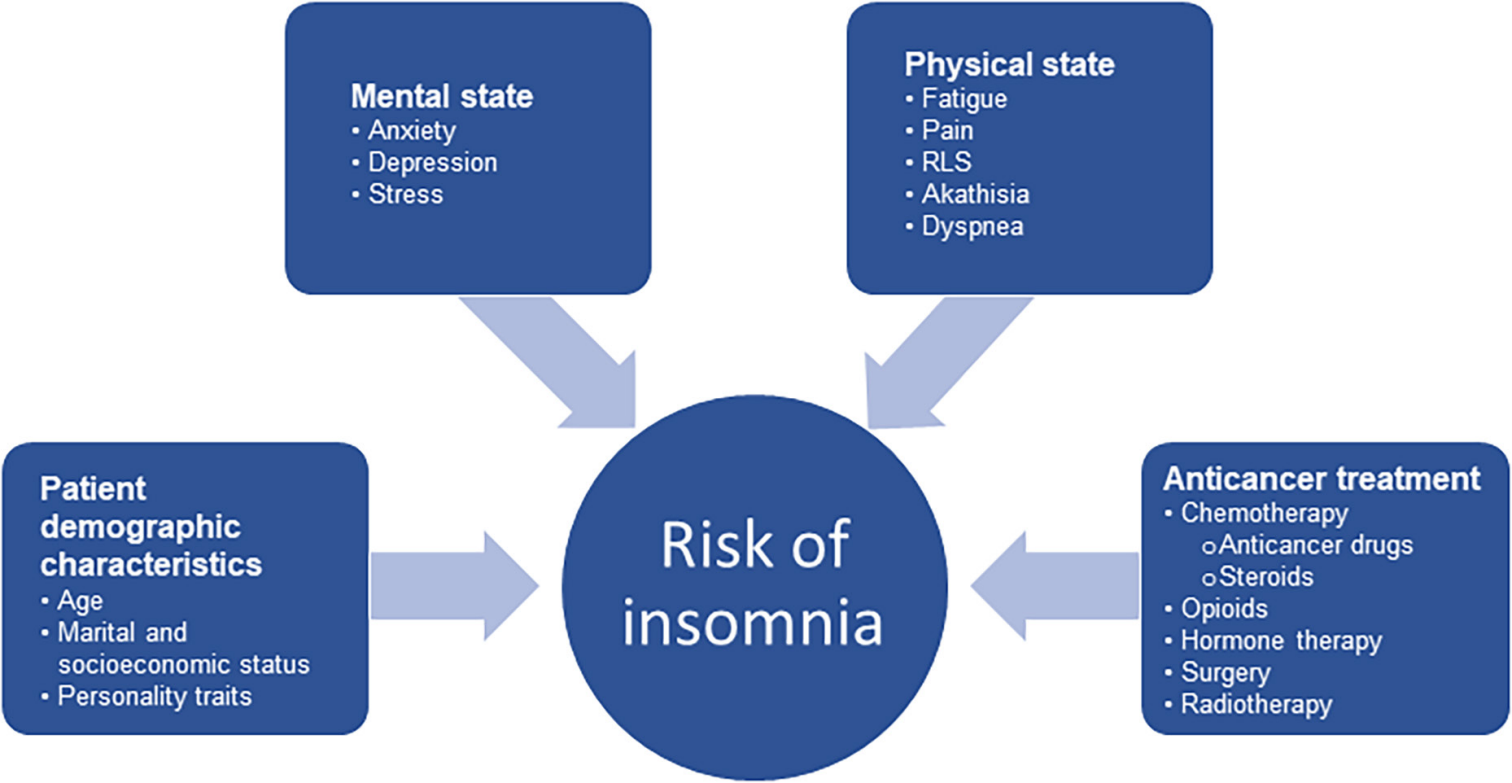
Factors associated with insomnia in patients with cancer. RLS, restless legs syndrome.

Based on the data reported in the articles we evaluated, we identified several factors associated with insomnia or sleep disturbance in patients with cancer and classified them into four categories (Figure 1): patient demographic characteristics, mental state, physical state, and anticancer treatment‐related. Below, we review these and other factors and propose potential management strategies for cancer‐related insomnia that take these factors into consideration.

### 4.1 Patient Demographic Characteristics Associated With Cancer‐Related Insomnia

The symptoms and conditions associated with insomnia in patients with cancer are multifactorial (Figure 1). Age was reported to be statistically significantly associated with insomnia in 15 studies; however, both younger age (eight studies) and older age (six studies) have been found to be statistically significant factors. Thus, the impact of age on the development of insomnia or sleep disturbance in patients with cancer warrants further investigation. In younger patients, it is possible that factors such as emotional distress, loss of societal roles, or financial challenges might contribute to sleep disturbances. For older patients, age‐related alterations in sleep patterns may make them more prone to insomnia [103]. Additionally, the potential influence of physical complications and the use of polypharmacy might also play a role in exacerbating sleep issues in the older age group.

### 4.2 Mental and Physical Factors Associated With Cancer‐Related Insomnia

Our research identified depression, anxiety, fatigue, and pain as frequent symptoms associated with insomnia in patients with cancer. This aligns with previous studies, in which statistically significantly higher frequencies of these conditions have been observed in patients with cancer who had insomnia [104, 105]. Moreover, these factors are intricately related, and cause and effect may be difficult to determine because several of these variables may induce insomnia or be induced by insomnia.

#### 4.2.1 Depression and Anxiety

In this analysis, depression was identified as the most frequent factor statistically significantly associated with cancer‐related insomnia (31/75 [41.3%] studies), and anxiety was identified as a statistically significant factor in 26 [34.7%] studies. Furthermore, in 19/75 (25.3%) studies, depression and anxiety were both reported to be statistically significant factors, indicating that these two conditions often co‐occur with insomnia in patients with cancer.

Insomnia is also one of the most frequently reported symptoms of depression [106], and there have been reports indicating a bidirectional relationship between insomnia and both depression and anxiety [107]. Insomnia may be exacerbated by depression and anxiety, thereby creating a deleterious feedback loop [108]. Therefore, early intervention for symptoms of insomnia, depression, and anxiety is considered necessary to contribute to the improvement of these conditions.

#### 4.2.2 Fatigue

Cancer‐related fatigue (CRF) is defined as “a distressing, persistent, subjective sense of physical, emotional, and/or cognitive tiredness or exhaustion related to cancer or cancer treatment that is not proportional to recent activity and interferes with usual functioning” [109], and has been reported to occur in more than 50% of patients with cancer [110]. In the present analysis, fatigue was frequently found to be statistically significantly associated with insomnia, including in patients with breast cancer (7/23 [30.4%] studies), gynecologic cancer (2/4 [50.0%] studies), brain cancer (2/4 [50.0%] studies), gastrointestinal cancer (4/9 [44.4%] studies), and prostate cancer (3/6 [50.0%] studies).

It is unclear whether insomnia is a constituent of CRF or occurs independently [111], but it has been suggested that elements of CRF, such as pain, depression, and poor concentration, are associated with sleep disturbances [112]. In addition, patients with breast cancer undergoing chemotherapy often show a symptom cluster of CRF–sleep disturbances–depression [113]. Thus, insomnia in patients with cancer may form a symptom cluster with pain, fatigue, and psychiatric conditions [6].

#### 4.2.3 Pain

Pain (including joint pain and neuropathic pain) was reported to be associated with insomnia in 24/75 (32.0%) studies in this analysis, with statistically significant associations commonly reported in breast cancer (7/23 [30.4%] studies), lung cancer (4/7 [57.1%] studies), and gastrointestinal cancer (4/9 [44.4%] studies). Pain can lead to sleep disturbances in patients with cancer, and conversely, sleep disturbances can enhance pain [114]. For example, in patients with breast cancer, disturbed sleep before radiotherapy has been reported to be associated with increased pain before, during, and after treatment [34], while the occurrence of neuropathic pain in patients with breast cancer has also been shown to be associated with a deterioration in sleep quality [25]. Furthermore, pain has a complex interrelationship with depression, fatigue, and anxiety.

#### 4.2.4 RLS and Mimics (Akathisia)

RLS is a sleep‐related sensorimotor disorder characterized by an urge to move, which occurs during rest or is exacerbated by rest, happens in the evening or night, and disappears or improves with movement [115]. The symptoms of RLS cause discomfort and can lead to insomnia. It has also been reported that the occurrence of RLS in patients with cancer is a factor that reduces their quality of life [116]. In our analysis, RLS was identified as a factor statistically significantly associated with insomnia or sleep disturbances in two studies [33, 84]. In patients with cancer undergoing chemotherapy, a direct relationship has been found between the PSQI score and the incidence of RLS; furthermore, recovery of RLS was associated with a statistically significant reduction in sleep disturbance [84]. These data indicate that RLS is a contributing factor to sleep disturbance in chemotherapy‐treated patients with cancer. In patients with early‐stage breast cancer treated with postoperative aromatase inhibitors, treatment significantly increased the prevalence and severity of RLS, and sleep quality was found to be statistically poorer in patients with RLS compared with patients without RLS [33].

Akathisia is a condition characterized by subjective discomfort; patients find it difficult to describe but may state that “it makes me want to run around the room or leap out of my bed” [117]. It is frequently drug‐induced and should be considered a sensorimotor disorder. Akathisia occurs commonly but often remains unnoticed in patients with cancer and results from the use of antiemetic medications during chemotherapy treatment [118], or from antipsychotic medications in delirium or palliative care settings [119]. In this patient population, akathisia may present as symptoms of anxiety and insomnia and may be difficult to identify and optimally manage [120].

### 4.3 Anticancer Treatments Associated With Cancer‐Related Insomnia

It is well known that chemotherapy can be associated with the development of insomnia. We identified one study in patients with early stage breast cancer that reported that the incidence of sleep disturbance was 36% before the start of chemotherapy, rising to 58% during treatment, and decreasing to 32.1% after chemotherapy ceased [22]. It has also been reported that the prevalence of insomnia increased in cervical cancer from 52.6% to 64.5% after adjuvant chemotherapy [47].

In patients with breast cancer receiving aromatase inhibitors, treatment was not reported to be associated with a statistically significant increase in insomnia prevalence [121]. However, it was noted that hot flashes often occur in patients receiving adjuvant aromatase inhibitors [121], and hot flashes have also been shown to be a risk factor for insomnia [21].

Associations between insomnia and other treatment modalities have also been reported. In one breast cancer study, patients receiving high doses of radiotherapy demonstrated a statistically significant decrease in sleep quality at 1 year [26]. The results of another observational study demonstrated a statistically significantly higher prevalence of sleep disturbances in patients with cancer who underwent surgery [89].

### 4.4 Management Proposals for Cancer‐Related Insomnia

**FIGURE 2:**
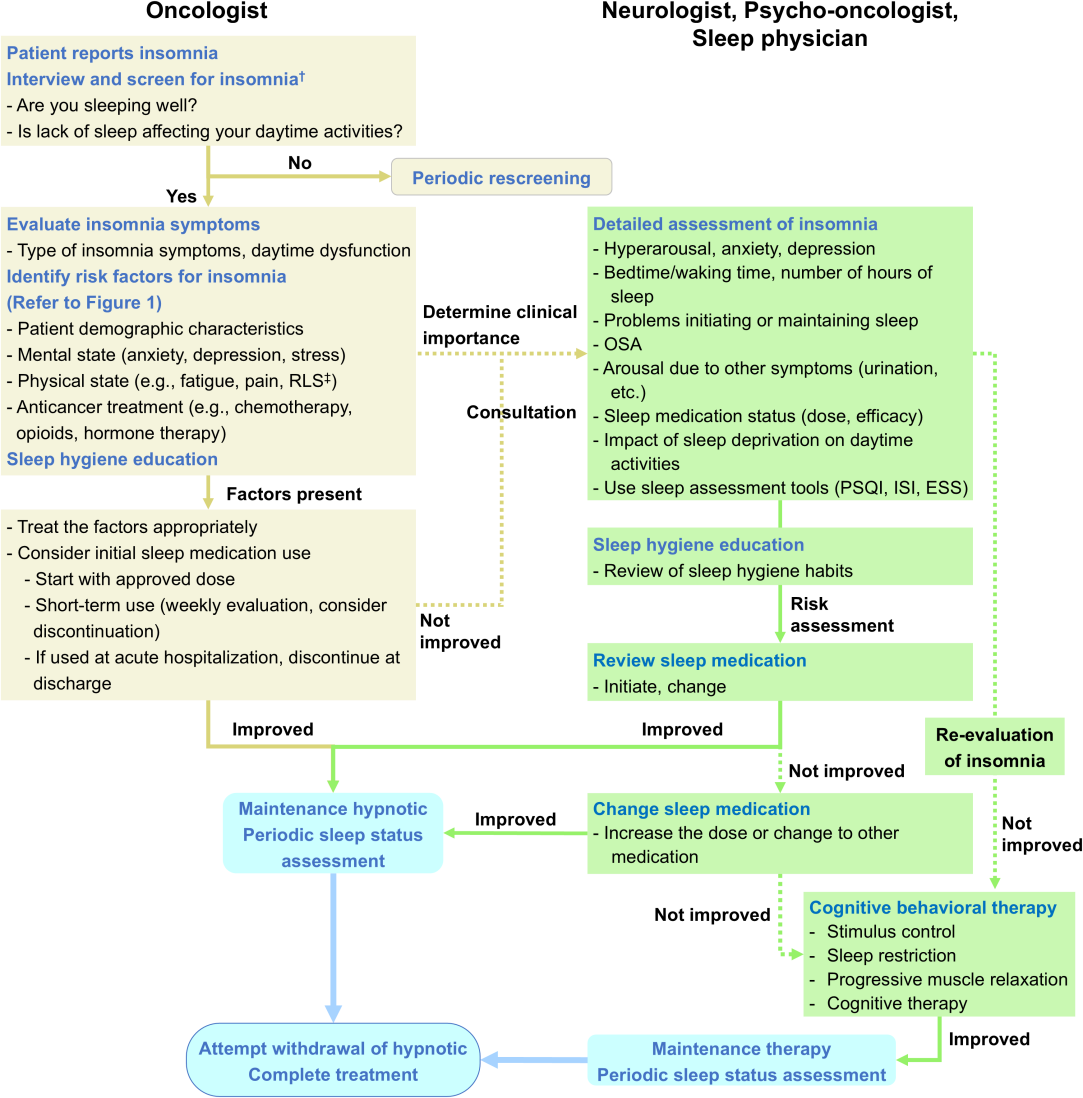
Suggestions for the management and treatment of insomnia in patients with cancer. ^†^As insomnia is often not reported by the patient, a detailed interview should be conducted. ^‡^Consider consulting a neurologist or psycho‐oncologist if RLS is severe. ESS, Epworth Sleepiness Scale; ISI, Insomnia Severity Index; OSA, obstructive sleep apnea; PSQI, Pittsburgh Sleep Quality Index; RLS, restless legs syndrome.

In patients with cancer, insomnia decreases the patient's quality of life and may result in the discontinuation of cancer treatment. As insomnia is often observed in association with other symptoms in patients with cancer, a comprehensive management strategy is required. We have therefore proposed a strategy to provide guidance for oncologists, with the aim of optimizing multidisciplinary interactions among medical professionals involved in the care of patients with cancer and insomnia, including neurologists, psycho‐oncologists, and sleep physicians (Figure 2).

#### 4.4.1 Screening of Insomnia

When evaluating insomnia in patients with cancer, it is important to understand that this condition may not be adequately diagnosed. Patients may not specifically report insomnia or may hesitate to do so until there is a severe impact on daily life [122], and physicians who are focused on cancer treatment may neglect to ask about comorbidities such as insomnia [123]. Thus, it is clear that interviewing the patient about their sleep quality throughout the cancer treatment period will improve the diagnostic rate for insomnia. Specifically, asking direct questions, such as “Are you sleeping well?” or “Is a lack of sleep impacting your daytime activities?,” is likely to be most effective [124], and it is desirable to ascertain sleep status by interviewing patients at an early stage after the start of cancer treatment.

#### 4.4.2 Assessment of Symptoms Associated With Insomnia

Our analysis indicates that several factors may be causally associated with insomnia, so it is important to properly assess items such as anxiety, pain, CRF, and RLS (Figure 2). If severe insomnia, depression, or anxiety are observed, physicians should consider consultation with a neurologist or psycho‐oncologist. Consultation should also be considered when insomnia persists despite non‐pharmacologic and initial pharmacologic therapy.

In addition to the factors identified in our literature review, attention must also be paid to whether patients with cancer‐related insomnia have OSA. OSA is a common sleep‐disordered breathing condition in which the throat narrows or collapses repeatedly during sleep, resulting in apneic events [125]. OSA produces intermittent hypoxia and frequent arousals, leading to an increase in respiratory effort, and is associated with cardiovascular comorbidities such as hypertension and ischemic heart disease. Its clinical signs include excessive daytime sleepiness, non‐restorative sleep, fatigue, or insomnia [125], and insomnia and sleep apnea are often comorbid, even among the general population [126]. Published evidence suggests that OSA occurs at noticeable levels among cancer patients in real‐world clinical practice and can lead to sleep disturbance and insomnia [127, 128, 129]. A recent meta‐analysis reported a 46% prevalence of OSA in patients with cancer [7], while two studies of patients newly diagnosed with lung cancer reported respective OSA prevalence rates of 49% and 80% [130, 131]. In both of the lung cancer studies, patients with OSA also reported daytime sleepiness, with respective Epworth Sleepiness Scale scores of 7.43 and 8.24 [130, 131]. Thus, if family members or caregivers report apnea or snoring in a patient with cancer, medical attention from a sleep physician should be sought.

Ideally, accurate assessment of cancer‐related insomnia should involve the use of both subjective measures like the PSQI and ISI, as well as objective measures such as PSG and actigraphy. However, consistently providing comprehensive assessments of sleep disorders can be burdensome, and both oncologists and patients may prefer a more concise method of evaluating insomnia. In the clinical setting, the validity of actigraphy has been demonstrated, with a test performance similar to that of PSG among people with insomnia [132], and this is one technique by which the evaluation of insomnia among patients with cancer may be improved. Although they are likely to be less burdensome for patients, the applicability of wearable devices as an objective tool for the assessment and treatment of insomnia in patients with cancer requires further investigation and validation [133].

#### 4.4.3 Non‐pharmacologic Therapy for Insomnia

We propose that physicians and healthcare teams should begin by initiating non‐pharmacologic therapies. It may also be useful to prepare educational materials and provide patients with information on insomnia and related issues.

Non‐pharmacologic therapies for insomnia in patients with cancer include a combination of sleep hygiene education, cognitive behavioral therapy‐insomnia (CBT‐I), and mindfulness‐based stress reduction (MBSR). Notably, compared with pharmacotherapy, CBT‐I may have long‐term beneficial effects for people with insomnia (with or without cancer or another comorbid condition) that persist after treatment ends [134, 135]. A meta‐analysis of insomnia in patients with breast cancer or survivors showed that CBT‐I was effective in reducing insomnia and improving sleep quality immediately post‐intervention, at 6 months, and at 12 months [11]. Furthermore, among cancer survivors (not restricted to breast cancer), CBT‐I has been reported to significantly improve the severity of insomnia and to show sustained benefits after 3 and 6 months of follow‐up [136]. The benefits of positive thought should not be underestimated in patients with cancer, as anxiety and insomnia are known to be linked [6], and two meta‐analyses have suggested that MBSR can help improve sleep quality in cancer survivors [14, 15]. It has also been suggested that statistically significant relief of sleep disturbances persists after cessation of MBSR and may reduce the use of benzodiazepine (BZD) sleep medications [137].

#### 4.4.4 Pharmacologic Therapy for Insomnia

If insomnia has not improved after a period of sleep hygiene education, pharmacologic therapy alone or in combination with CBT should be considered. In our research, melatonin was commonly used for the treatment of cancer‐related insomnia (Table 2), and a recent systematic review of the impact of melatonin on sleep quality and insomnia in patients with cancer found statistically significant effects, suggesting that melatonin may have some efficacy in this patient population [138]. However, each melatonin study was small, and the use of melatonin to treat insomnia in patients with cancer requires further robust clinical evaluation. Therefore, at the current time, the use of melatonin as an aid to sleep in cancer patients with insomnia is not recommended.

In a single‐blind study with a melatonin comparator, zolpidem was reported to be effective for insomnia relief in patients with colorectal cancer [97], and both drugs were found to statistically significantly improve sleep quality (Table 2). In another study, zolpidem statistically significantly improved PSQI scores in patients with solid tumors [98]. In a placebo‐controlled study of zopiclone in patients with advanced cancer, self‐reported sleep quality and sleep onset latency were statistically significantly improved in zopiclone‐treated patients compared with the placebo group [100]. However, BZDs and BZD receptor agonists such as zolpidem, while commonly used, should not be the first choice for pharmacologic treatment of insomnia in patients with cancer. These drugs can trigger delirium [139, 140], and have been reported as a risk factor for developing delirium in patients with cancer [141]. In addition, adverse events such as cognitive dysfunction [142], falling [143], development of tolerance and dependence [144, 145], and breathing disorders [146] have been reported to be associated with high doses and long‐term use of these drugs, and these symptoms negatively affect treatment continuation and quality of life for patients with cancer. Therefore, BZDs should be used with caution to treat cancer‐associated insomnia, and the potential for adverse events should be considered when prescribing them.

The recently approved class of DORAs are considered to be safer drugs than BZDs and BZD receptor agonists and are believed to induce physiological sleep by suppressing the function of the arousal center [147]. A recent meta‐analysis reported that the DORAs suvorexant and lemborexant are efficacious and safe for patients with insomnia [148]. However, data on their use in patients with cancer are lacking; to date, one report on lemborexant in patients with cancer has been published (Table 2). In a retrospective observational study in patients with insomnia with delirium, lemborexant was administered instead of, or in combination with, other psychotropic agents; lemborexant was reported to be effective in 11/14 (78.6%) patients with insomnia [102]. In addition, in a retrospective study of patients with pancreato‐biliary disease (*n* = 64) who developed insomnia after an endoscopy, the time to fall asleep was successfully reduced in 61/64 (95.3%) patients by administering lemborexant without increasing the risk of delirium [149]. There are also reports of lemborexant and suvorexant being safely used to treat OSA [150, 151, 152], a condition that affects almost half of patients with cancer [7]. Overall, DORAs have advantages over conventional hypnotics in that they do not cause delirium and cause less respiratory depression, but owing to the paucity of published data on their use in patients with cancer, further studies are clearly needed. Of note, the prospective observational LUNAR study is currently underway to evaluate the efficacy of lemborexant in patients with breast cancer who have insomnia (UMIN000047718). For the recently approved DORA daridorexant, there are currently no published data in patients with cancer, and clinical studies are needed to evaluate its efficacy in this population.

Trazodone is an antidepressant classified as a serotonin antagonist and reuptake inhibitor. Over the past 20 years, its use has shifted towards treating insomnia at lower doses (25–100 mg) rather than being prescribed as an antidepressant. The mechanism through which it produces its hypnotic effect is via blockade of the 5‐HT_2A_, histamine H1, and alpha1 adrenergic receptors [153]. However, recent network meta‐analyses on insomnia pharmacotherapy indicate that while trazodone may alleviate acute insomnia symptoms, its tolerability remains a concern [154]. Reports on trazodone's efficacy for insomnia in patients with cancer are also limited (Table 2) [98], and further research is needed to assess its respective benefits and harms in this population.

#### 4.4.5 Treatment of Comorbid Symptoms With Insomnia

As previously mentioned, insomnia forms clusters with other symptoms, such as depression, anxiety, fatigue, and pain, with each symptom influencing others. Thus, patients should be referred to a neurologist or psycho‐oncologist for appropriate management of these symptoms (Figure 2). Consultation with a neurologist or psycho‐oncologist should also be considered when RLS is observed and with a sleep physician when OSA is present. For pain, appropriate analgesics should be prescribed. If fatigue associated with cancer treatment is observed, dose reduction should be considered where possible [155].

### 4.5 Study Limitations

There are many potential limitations that must be considered when evaluating these results. The purpose of this study was to identify factors associated with cancer‐related insomnia and to propose management strategies; as we did not intend to formally assess diagnosis, prognosis, or interventions, the search protocol was not pre‐registered in PROSPERO. In addition, because this review aimed to investigate trends in cancer‐related insomnia research, we did not assess the risk of bias in each of the included studies. There is also the possibility that some studies were not included in the literature search despite the use of two databases; criteria such as the inclusion only of studies published in English may have resulted in studies published in other languages being omitted. We also excluded non‐pharmacologic interventions for sleep disturbance from our analysis.

In addition, the identification of considerable heterogeneity among studies limits the inferences that can be made. Studies varied in their definition and measurement of insomnia or sleep disturbance severity, the use of different scales and cut‐off values, and observation periods and evaluation timepoints. Participant background characteristics, such as cancer type/stage and cancer therapy, also varied widely, as did the methods used to determine risk factors for insomnia. Indeed, this broad heterogeneity among studies meant that we chose not to perform specific data integration for the factors we categorized as being associated with insomnia in patients with cancer, as we judged that the results would not be sufficiently robust. However, the information presented in Table 1, which describes the design and results from each included study, serves to indicate potential areas warranting further investigation, such as meta‐analyses focusing on each of the identified risk factors.

Despite these limitations, we believe that this review delivers meaningful information for clinicians in that it provides a broad survey of relevant studies and furnishes themes for future research.

### 4.6 Clinical Implications

In this study, we reviewed the prevalence of insomnia in patients with solid tumors and found that it remains a major clinical issue, affecting between 14.8% and 81.5% of patients. In addition, anxiety, depression, fatigue, and pain were identified as factors that may induce insomnia or as symptoms that are likely to occur in association with insomnia, suggesting that these disorders often form clusters.

Insomnia tends to be overlooked and underestimated during cancer treatment, likely because physicians are not aware of its impact on treatment and patients are reluctant to report it. Therefore, we have proposed guidance for efficient screening and appropriate management, including consultation with neurologists and psycho‐oncologists, and clarified the role of the oncologist in the treatment of insomnia. Improving awareness within the healthcare team about insomnia, its cluster syndromes, and its treatment in patients with cancer will drive improvements in the quality of cancer care. Overall, it is important for the healthcare team to consider the importance of insomnia and associated symptoms and to provide optimal management for patients with cancer.

### 4.7 Conclusions

The results of our systematic literature evaluation indicate that a large proportion of patients with cancers of many types are affected by insomnia or sleep disturbance, and enabled us to identify some potential risk factors for developing insomnia among this patient population. Pharmacologic treatment specifically for cancer‐related insomnia appears to be suboptimal, with large evidence gaps. Taking these factors into consideration, we have proposed potential intervention and management pathways to better optimize medical care for affected patients.

## Author Contributions

**Yoshinori Saeki:** conceptualization (equal), data curation (supporting), investigation (equal), methodology (equal), project administration (lead), validation (supporting), visualization (equal), writing – original draft (equal), writing – review and editing (equal). **Yukiyoshi Sumi:** conceptualization (equal), data curation (lead), investigation (equal), methodology (equal), project administration (supporting), software (lead), validation (lead), visualization (equal), writing – original draft (equal), writing – review and editing (equal). **Yukinori Ozaki:** conceptualization (supporting), supervision (supporting), writing – review and editing (supporting). **Mari Hosonaga:** conceptualization (supporting), supervision (supporting), writing – review and editing (supporting). **Yoshiyuki Kenmotsu:** conceptualization (supporting), supervision (supporting), writing – review and editing (supporting). **Takuma Onoe:** conceptualization (supporting), supervision (supporting), writing – review and editing (supporting). **Ken Yamaguchi:** conceptualization (supporting), supervision (supporting), writing – review and editing (supporting). **Yoshihisa Matsumoto:** conceptualization (equal), methodology (equal), project administration (supporting), supervision (lead), visualization (equal), writing – original draft (supporting), writing – review and editing (equal).

## Conflicts of Interest

Although authors received payment for advisory board services, this present manuscript was prepared independently with funding for medical writing only. Yukiyoshi Sumi received funding for the present manuscript from Eisai Co. Ltd., and received payment or honoraria for lectures or presentations from MSD K.K., Sumitomo Pharma Co. Ltd., Kyowa Kirin Co. Ltd., Viatris Pharmaceuticals Japan Inc., Takeda Pharmaceutical Co. Ltd., and Eisai Co. Ltd. Yukinori Ozaki received payment or honoraria for lectures, presentations, speakers' bureaus, manuscript writing, or educational events from Daiichi Sankyo Co. Ltd., Pfizer Japan Inc., Kyowa Kirin Co. Ltd., and Eli Lilly Japan K.K. Yoshiyuki Kenmotsu received funding for the present manuscript, payment or honoraria for lectures, payment for expert testimony, and payment for buiness meetings from Eisai Co. Ltd.; payment or honoraria for lectures from Chugai Pharmaceutical Co. Ltd., AstraZeneca K.K., Takeda Pharmaceutical Co. Ltd., KYORIN Pharmaceutical Co. Ltd., Eli Lilly Japan K.K., Janssen Pharmaceutical K.K., and Nippon Boehringer Ingelheim Co. Ltd. TO received funding for the present manuscript, grants to the author's institution, and consulting fees from Eisai Co. Ltd. and payment or honoraria for lectures, presentations, speakers' bureaus, manuscript writing, or educational events from Eisai Co. Ltd., Chugai Pharmaceutical Co. Ltd., Taiho Pharmaceutical Co. Ltd., and Bristol‐Myers Squibb K.K. KY received funding for the present manuscript from Eisai Co. Ltd.; grants, consulting fees, and payment associated with a patent held by DUMSCO Inc.; payment or honoraria for lectures, presentations, speakers' bureaus, manuscript writing, or educational events from Eisai Co. Ltd., Takeda Pharmaceutical Co. Ltd., Chugai Pharmaceutical Co. Ltd., Daiichi Sankyo Co. Ltd., and Merck & Co. Inc.; and served a leadership or fiduciary role (unpaid) in the Japan Society of Obstetrics and Gynecology and Japan Society of Endometriosis. YM received funding for the present manuscript from Eisai Co. Ltd. Yukinori Ozaki and Mari Hosonaga declare no conflicts of interest.

## Supporting information


**Data S1** Literature search and screening strategy.
**Figure S1.** PRISMA diagram of the systematic literature review for the evaluation of risk factors.

## Data Availability

Data sharing is not applicable to this article as no datasets were generated or analyzed in this study.
