# Supplementary material for: Proposal for Managing Cancer‐Related Insomnia: A Systematic Literature Review of Associated Factors and a Narrative Review of Treatment
Source: Cancer Med. 2024 Nov 25;13(22):e70365. doi: 10.1002/cam4.70365 (PMC11586868; doi:10.1002/cam4.70365)
Supplement: Supplementary file 1 — Data S1 Literature search and screening strategy. Figure S1. PRISMA diagram of the systematic literature review for the evaluation of risk factors. [file CAM4-13-e70365-s001.docx]

**Supplementary information**

**Supplementary Text 1. Literature search and screening strategy**

**Supplementary Figure 1. PRISMA diagram of the systematic literature review for the evaluation of risk factors**

**Supplementary Text 1. Literature search and screening strategy**

***Literature searches***

The search string for the PubMed database was as follows: (sleep disorder*[TIAB] OR sleep disturbance[TIAB] OR sleep quality[TIAB] OR insomnia[TIAB] OR suvorexant[TIAB] OR lemborexant[TIAB] OR benzodiazepine[TIAB] OR brotizolam[TIAB] OR lormetazepam[TIAB] OR zolpidem[TIAB] OR zopiclone[TIAB] OR eszopiclone[TIAB] OR melatonin[TIAB] OR ramelteon[TIAB] OR GABA receptor antagonist*[TIAB] OR sleeping pill*[TIAB] OR hypnotic*[TIAB]) AND (cancer[TIAB] OR tumor[TIAB] OR adenocarcinoma[TIAB] OR neoplasm[TIAB]) AND ("2012"[CRDT] : "3000"[CRDT]).

The search string for the Scopus database was as follows: (TITLE (sleep disorder) OR TITLE (sleep disturbance) OR TITLE-ABS ("sleep disorder") OR TITLE-ABS ("sleep disturbance") OR TITLE-ABS ("sleep quality") OR TITLE-ABS (insomnia) OR TITLE-ABS (suvorexant ) OR TITLE-ABS (lemborexant) OR TITLE-ABS (benzodiazepine) OR TITLE-ABS (brotizolam) OR TITLE-ABS (lormetazepam) OR TITLE-ABS (zolpidem) OR TITLE-ABS (zopiclone) OR TITLE-ABS (eszopiclone) OR TITLE-ABS (melatonin) OR TITLE-ABS (ramelteon) OR TITLE-ABS (GABA receptor antagonist*) OR TITLE-ABS (sleeping pill*) OR TITLE-ABS (hypnotic*)) AND (TITLE-ABS (cancer) OR TITLE-ABS (tumor) OR TITLE-ABS (adenocarcinoma) OR TITLE-ABS (neoplasm)) AND PUBYEAR > 2011.

***Screening for frequency of insomnia in patients with cancer***

The inclusion criteria for this step were as follows: include “sleep/insomnia” AND “cancer/tumor/carcinoma” in the Title, AND include [“any psychiatric symptoms (fatigue, cognitive, depression, anxiety)” OR “any assessment scale (polysomnography, DSM-5, ICSD-3, Insomnia Severity Index, ISI, PSQI, AIS, Epworth Sleepiness Scale, EORTC QLQ C-30)” OR “sleep quality” OR “any hypnotic”] in Title/Abstract.

Exclusion criteria were any of the following cancer types “head and neck, brain, prostate, bladder, colorectal, colon, rectal, gastric, gastrointestinal, digestive” in the Title, OR “survivor, cognitive behavioral, mindfulness” in the Title, OR “palliative, acupuncture, acupressure, herbal, aromatherapy” in the Title/Abstract.

***Screening for the efficacy of hypnotics for insomnia in patients with cancer***

The inclusion criteria for this step were as follows: include “sleep/insomnia” AND “cancer/tumor/carcinoma” in the Title, AND include hypnotics “benzodiazepine, **zepam, *zolam, gamma amino butyric acid, GABA-A, zolpidem, zopiclone, eszopiclone, ramelteon, melatonin, orexin, suvorexant, lemborexant, hypnotic, sleeping pill” in the Title/Abstract.

**Supplementary Figure 1. PRISMA diagram of the systematic literature review for the evaluation of risk factors**

**
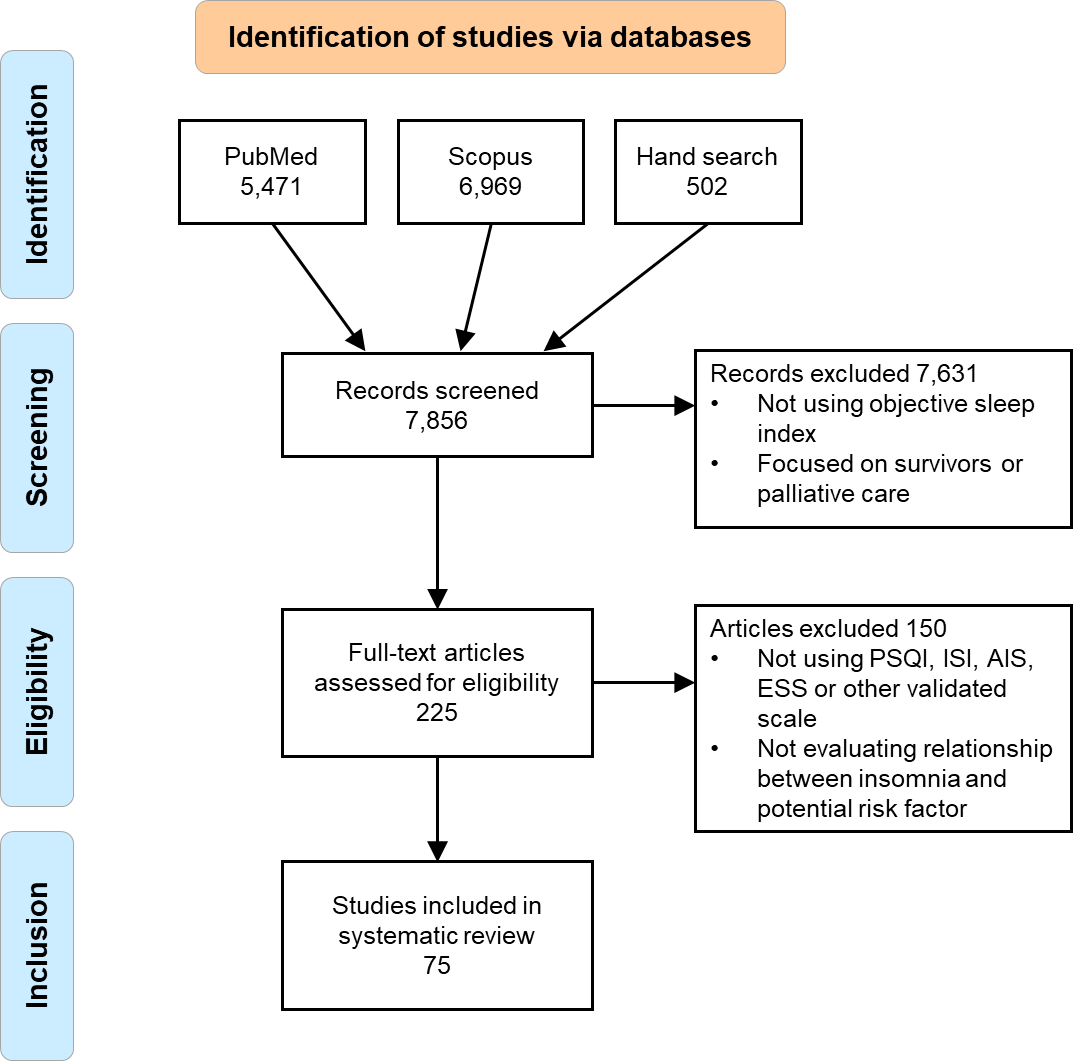
**

AIS, Athens Insomnia Scale; ESS, Epworth Sleepiness Scale; ISI, Insomnia Severity Index; PRISMA, Preferred Reporting Items for Systematic Reviews and Meta-Analyses; PSQI, Pittsburgh Sleep Quality Index.
